# Now you see it, now you don’t: Relevance of threat enhances social anxiety-linked attentional bias to angry faces, but relevance of neutral information attenuates it

**DOI:** 10.1371/journal.pone.0271752

**Published:** 2022-07-28

**Authors:** Julia Vogt, Helen F. Dodd, Alice Parker, Francesca Duffield, Michiko Sakaki

**Affiliations:** 1 School of Psychology and CLS, University of Reading, Reading, United Kingdom; 2 College of Medicine and Health, University of Exeter, Exeter, United Kingdom; University of Amsterdam, NETHERLANDS

## Abstract

Temporary goals modulate attention to threat. We examined whether attentional bias to angry faces differs depending on whether a temporary background goal is neutral, or threat related, whilst also measuring social anxiety. Participants performed a dot probe task combined with a separate task that induced a temporary goal. Depending on the phase in this goal task, the goal made angry faces or neutral stimuli (i.e., houses) relevant. The dot probe task measured attention to combinations of angry faces, neutral but goal-relevant stimuli (i.e., houses), and neutral control stimuli. Attention was allocated to angry faces when an angry goal was active. This was more pronounced for people scoring high on social phobia. The neutral goal attenuated attention to angry faces and effects of social phobia were no longer apparent. These findings suggest that individual differences in social anxiety interact with current and temporary goals to affect attentional processes.

## Introduction

People attend to various emotional and motivationally relevant events [1]. For instance, individuals high in anxiety show an attentional bias toward threat [2]. Even temporary goals and needs impact attention. For example, hungry observers allocate attention to food [3]. Here, we investigate the impact of temporary goals on attention to threatening stimuli, that is, angry faces, by varying whether participants pursue a threat-related or neutral background goal when performing the attention task. This will allow us to understand how temporary goals impact attention to threat and, by measuring individual differences in social anxiety, whether such effects differ depending on level of social anxiety.

Many theories advocate a special attentional status of threat, specifically, that attention to threat is automatic to ensure survival [4]. Supporting this assumption, rare fearful faces cause larger attentional orienting than rare happy faces [5; see also 6]. Such findings suggest that threat attracts attention independent of top-down settings, that is, even when it is neither expected nor relevant to any temporary goal. Prominent models of emotional attention therefore emphasize that emotional events can attract attention in a bottom-up driven manner [4]. However, these models have been challenged by recent studies that failed to find such automatic attention to threat in non-anxious samples [e.g., 7,8].

In a separate line of research, attention to threat has been more reliably found when individuals are high in fear and anxiety despite some inconsistency in findings [see 9]. For instance, in a meta-analysis of 172 studies, threat does not bias attention in non-anxious controls but does bias attention in anxious participants [2]. Attention to threat is found across specific anxiety disorders including social phobia [10, 11]. Therefore, attentional bias to threat has been discussed as a factor that contributes to the development and maintenance of anxiety and phobias [2, 12–14]. However, there is still a debate about whether enhanced attention to threat, associated with anxiety, is due to top-down or bottom-up processes.

Attentional biases are not limited to emotional events. The pursuit of temporary goals causes an attentional bias to goal-relevant stimuli even when such stimuli are neutral and without a history of motivational relevance to the observer or our species [15, 16]. This work is in line with theories suggesting that the attentional system supports the preparation and execution of actions and current goals [17]. Goal-relevant information attracts attention rapidly and unintentionally, suggesting this process is relatively automatic, even though it is influenced by top-down goals [15–20].

The pursuit of temporary but threat-unrelated goals appears to attenuate attentional bias to threat, especially when threatening events are presented together with neutral events that are relevant to such a temporary goal [21–23]. For instance, in [23, see also 24], researchers presented observers with threat-related and neutral stimuli in a dot probe paradigm. Some of the neutral stimuli were relevant to a temporary goal of winning tokens in a secondary task. Threatening events attracted attention when they were presented with neutral stimuli that were not relevant to the goal. However, people attended to neutral but goal-relevant stimuli when they were presented together with threatening stimuli, even in a sample of high trait anxious people and when using imminent threat (i.e., stimuli signalling the delivery of loud white noise). However, the effect has been replicated only in unselected samples [e.g., 24]; therefore, it remains unclear whether neutral goal-relevant stimuli can override attention to threat in individuals who have high levels of anxiety or phobias.

When the focus of a temporary goal is threat-related, this enhances attention to threat even in individuals who are not highly anxious. For instance, threat-related faces pop out when participants have a goal of searching for them [25]. Interestingly, this ‘pop-out’ effect does not occur for threat-unrelated faces. Further, when they are relevant to temporary goals, angry faces [26, 27], fearful faces [28], and spiders [29] have all been shown to evoke an attentional bias, even in non-anxious participants [8, 30]. Consequently, differences in attention to threat between anxious and non-anxious participants are erased when task goals require all participants to search for emotional faces [31]. Some authors have therefore argued that enhanced attention to threat in fear and anxiety may be underpinned, at least to some extent, by top-down factors such as current threat-related goals or expectations [32–37] that are continuously activated in anxious individuals.

In the present study, we extend this work to the effects of social anxiety on attention to angry faces. Although social anxiety is one of the most common types of anxiety experienced, most of the research in this area has so far measured trait anxiety, rather than focusing on social anxiety specifically [27]. Importantly, individual differences in social anxiety may have distinct effects on attention to threat depending on whether threat is goal-relevant or goal-irrelevant. The preceding evidence suggests that differences between anxious and non-anxious participants will become evident when emotion is not relevant to temporary goals. Indeed, in previous research, individual differences in social anxiety influenced attention to emotional faces only when the angry face was not related to the goal via impairing shifts to happy faces [27; see also 31, 38, 39]. Social anxiety might thus not enhance the effects of threat-related goals on attention but may cause attentional disruption when participants are asked to pursue a threat-unrelated goal or task in the presence of threat [but see 23].

Alternatively, individual differences in social anxiety might emerge when threat is relevant, via anxiety promoting the adoption of threat-related goals, for instance, because threat-related goals are more motivating for anxious compared to non-anxious participants or easier to adopt because they reflect anxious observers’ attentional selection history [14]. Existing studies have not enabled this to be sufficiently evaluated, primarily because inducing a very specific goal to allocate attention to angry faces will produce a strong attentional bias in all participants that cannot be further augmented by individual differences in anxiety [8, 20]. In contrast, differences in motivational strength within the context of goal pursuit can be observed when relevance is induced via a background goal in a separate task rather than by directly instructing attention to goal-relevant stimuli [16], which is the approach we adopt here.

### The present research

In the present paper, we will investigate how temporary neutral and threat-related background goals that are pursued in a secondary task impact attention to threat. Our study will test whether neutral goals prevent attention to angry faces even in socially anxious observers and whether the impact of threat-related goals on attention to angry faces is enhanced in socially anxious observers. Our study will contribute to the debate whether attention to threat is automatic (i.e., angry faces attract attention even when not goal relevant) or is caused by relevant top-down goals (i.e., angry faces bias attention when they are relevant to participants’ background goal; see [2, 8]).

To test the attentional biases for threatening and neutral events that are either goal-relevant or goal-irrelevant, we combined an attention paradigm with a secondary task that made either threatening (i.e., angry faces) or neutral stimuli (i.e., houses) goal relevant in this task [cf. 16, 23]. Participants alternated between neutral and threat-related goals in different blocks to allow us to compare the effects of different temporary goals in the same participants. To test the attentional processing of both types of stimuli when they were goal relevant and goal-irrelevant to the secondary task, we presented different angry faces and houses as cues in a dot probe paradigm, paired with control stimuli and with each other, in between trials of the goal task. Thus, our dot probe paradigm included three types of pairs: a) angry faces vs. control pairs (i.e., threat-control pairs), b) houses vs. control pairs (i.e., house-control pairs), and c) angry faces vs. houses pairs (i.e., threat-house pairs). Each pair was followed by a dot shown at the location of one of the images; participants were asked to indicate the location of the dot as quickly as possible.

We hypothesize that threatening stimuli will attract attention when presented together with neutral control stimuli, even when houses are goal relevant [23, 40]; in other words, we expect that for threat-control pairs, participants would be faster to react to the dot when it appears behind an angry face than a control stimulus both in the house goal phase and the angry-face goal phase. However, in house goal phases, attention to threat will be attenuated in the presence of goal stimuli (i.e., houses). Further, we expect house stimuli to attract attention when houses are goal relevant and are presented together with control stimuli. We will also explore the impact of social anxiety on attention to threat to see whether social anxiety enhances attention to threat when threat is relevant to the current goal or not, or both.

## Materials and methods

### Participants

One-hundred and seventy-eight students (23 men; *M*_*age*_ = 21.3 years, *SD*_*age*_ = 6.79) took part in exchange for course credit. All participants had normal or corrected-to-normal vision. None were currently taking prescription medication for anxiety or depression. Sample size was determined to be a minimum of 160 participants based on the effect size of *d* = .45 reported in the meta-analysis by [2] at an alpha of 0.05 and power at 0.8 to be able to detect an attentional bias to threat and differences between participants based on anxiety. Ethical approval was granted by the School of Psychology and CLS at the University of Reading.

### Apparatus and materials

#### Images

Goal-relevant images consisted of 20 angry faces (10 men, 10 women), and 20 images of houses/buildings (10 each). Half of the angry faces (5 men; 5 women) were used in the dot probe task, while the others were used in the goal task. Likewise, half of the images of buildings/houses (5 buildings; 5 houses) were used in the dot probe task, while the others were used in the goal task. Making a category instead of single stimuli goal relevant and using different sets of stimuli in the tasks will test whether goal-driven effects arise beyond specific stimuli that participants have learned to be goal relevant and thus permits greater generalizability. We also used 20 images of cars as control stimuli; half for the dot-probe task and half for the goal task. We used car stimuli to have a neutral stimulus category of similar complexity. The stimuli used in the dot-probe task and goal task were counterbalanced across participants. We further used 10 neutral faces in the goal task to prevent participants from not paying attention to the facial expression.

Face stimuli were taken from the Karolinska Directed Emotional Faces database [41]. Faces were selected based on validity ratings [42]. We did not use faces with open mouths and used different models for angry and neutral faces to eliminate familiarity effects. Images of houses and buildings were taken from those used in previous studies [43, 44]. Images of car were selected following online searches. We chose them because the visual complexity is similar to faces [cf. 45]. All images were presented in greyscale and sized 5.1 cm x 5.1.cm.

#### Questionnaires

The Social Phobia Inventory (SPIN; [46]) was used to measure social anxiety. It consists of 17 questions which focus on three main components of social anxiety: fear (e.g., of people in authority, of social events), avoidance (of specific social situations), and physiological responses (e.g., blushing, sweating, trembling). Items are rated on a 5-point Likert scale with higher scores indicating greater distress.

The brief version of the Fear of Negative Evaluation Scale was also used (BFNE; [47]). This consists of 12 statements (e.g., I am frequently afraid of other people noticing my shortcomings), with the participant asked to write how characteristic each statement is of them on a 5-point Likert scale with five indicating the most characteristic.

The State and Trait Anxiety Inventory [48] was used to measure participant’s overall anxiety levels. This is subdivided into two separate questionnaires, each of these consisting of 20 statements beginning with ‘I feel’, which the participant rates on a 4- point Likert scale (1 = *‘not at all’*, 4 = *‘very much so’*). The trait anxiety questionnaire (TAI) asks participants to consider how they generally feel, whereas the state (SAI) questionnaire assesses anxiety levels at the present time.

To assess each participant’s overall emotional state, the Positive Affect and Negative Affect scale (PANAS; [49]) was used. This is comprised of 20 descriptive emotional terms, and the participant is asked to rate the extent to which they feel this way on a 5-point Likert scale with five indicating they feel that emotion ‘extremely’. Questionnaires were presented in the order as described here.

We also asked for various demographics including age, gender, years of received education, ethnicity, profession, sleep patterns, self-rated stress and health levels, first language, handedness, day in menstrual cycle, and medication participants were currently taking.

#### Dot probe and goal inducing task

**Fig 1:**
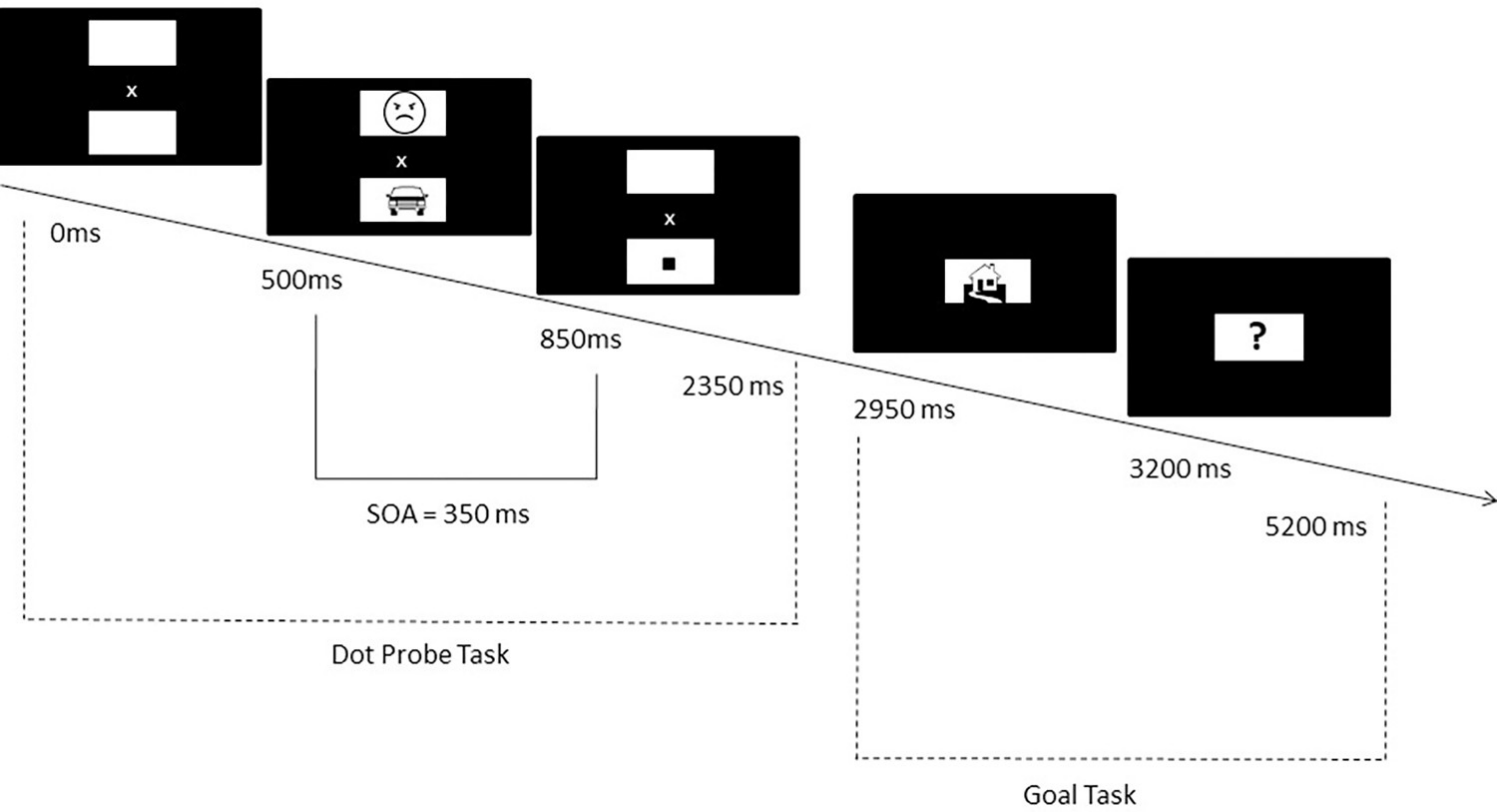
Schematic overview of a trial of the combined dot probe and goal task. The first three boxes depict the dot probe task in which the presentation of the cues was followed by a probe (black square) which had to be localized. Cues and goal stimuli were photographs in the study. The last two boxes display the goal task in which the presentation of a single stimulus was followed by the appearance of a question mark. Participants had to react to the question mark by pressing the spacebar when the single stimulus presented had been goal stimulus. A goal trial only followed a trial in the dot probe task from time to time.

Participants alternated between trials of the dot probe and goal-inducing task. The goal in the latter task varied between angry faces and houses in different blocks of the study to test the effect of these two background goals on attention allocation. Each *dot probe* trial began with the presentation of a white fixation cross (5 mm high) in the centre of a grey background (see Fig 1; please note that cues and goal stimuli were photographs in the study). Two pale grey squares (5.1 cm x 5.1 cm) were presented above and below the fixation cross. The distance between the middle of each square and the cross was 4.6 cm. After 500 ms, two image cues replaced the squares for 350 ms [cf. 23]. Hereafter, a black dot (0.9 cm x 0.9 cm) was presented in the centre of one of two pale grey squares. Participants had to press the ‘4’ key on the number pad for the upper location and the ‘5’ key for the lower location. If no response was given, the program proceeded after 1500 ms.

After 600 ms of a trial in the attention task, a trial of the *goal inducing task* started with the presentation of an image in the centre of the screen for 250 ms. It was replaced by a red question mark on a pale grey square (5.1.cm x 5.1 cm). Participants had to press the spacebar only if the preceding image was goal relevant. Specifically, when participants were in the threat-related goal phase they were asked to press the spacebar if the image was of an angry face, whereas when they were in the threat-unrelated goal phase they were asked to press the spacebar only when the image was a house. In both conditions, if no response was given, the program proceeded after 2000 ms. A feedback message stating ‘Correct! +1’ in lime green or ‘Incorrect! +0’ in yellow was presented for 200 ms.

### Procedure

The tasks were presented using E-Prime 2.0 (E-Studio and E-Run; [50]) on a Dell Optiplex 9020 PC. Participants were seated approximately 60 cm from a computer screen. The test procedure began with gaining informed consent, and the collection of demographic information.

Then, participants first practiced the dot probe task (without the goal task) in six trials. They were asked to maintain attention at the fixation cross and to respond as quickly and as accurately as possible to the probe location. Participants next completed another practice phase, where they practiced the goal task (without the dot probe task). This second practice phase included five trials for a threat-related goal (i.e., angry faces) and another five trials for a threat-unrelated goal (i.e., houses). Instructions informed participants that a single picture would be presented in the middle of the screen. The instructions specified that if this picture belonged to the goal-relevant category (i.e., angry faces or houses), they should press the spacebar with the left hand when the question mark appeared. Instructions further informed participants that they would win one point for correctly indicating the presence of the goal-relevant picture during the goal task. Participants next practiced both the dot probe task and the goal task together as in the main test phase; we included five trials for the threat-related goal and another five trials for the threat-unrelated goal. Stimuli used in the practice trials were different from those used in the main test phase.

During the main test phase, participants alternated between blocks with a threat-related goal and blocks with a threat-unrelated/ neutral goal. There were four blocks. Half of the participants completed the angry-face goal block first followed by the house goal block, while the other half of participants completed the house goal block first followed by the angry-face goal block. Participants continued to alternate between the two goals. The goal order was counterbalanced across participants. Each block included 60 trials of the dot probe task and 21 goal task trials (six goal-relevant trials, 15 filler trials with cars). These 81 trials were presented in a random order, determined for each participant. Trials in the dot probe task were angry faces vs. houses, angry faces vs. cars, and houses vs. cars. Each picture category was presented on half of the trials in the upper cue location and on the other half in the lower cue location. For each picture category, the probe was presented on the location of that picture category on half of the trials.

Finally, participants completed the questionnaires detailed above to assess social anxiety and broader anxiety and affect. Hereafter, they were fully debriefed, and given the opportunity to ask questions about the research.

## Results

Trials with errors were excluded from analyses (4.58%). Reaction times faster than 150 ms and slower than 1000 ms (0.75%) were excluded from the analyses in line with [23]. As a robustness check, we repeated the analyses using the medians of the RTs. Analyses came to the same conclusions with minor exceptions that we will note. The data of three participants were not recorded at all due to due to technical difficulties, the data of a fourth participant were excluded as less than 10 trials were recorded. To perform additional analyses, we calculated attentional bias scores by subtracting mean RTs on congruent trials from mean RTs on incongruent trials. Means and standard deviations for RTs can be found in Table 1. The data set can be found at: https://osf.io/rt9n2/.

**Table 1:** Mean RTs and standard deviations (in ms) as a function of phase goal, trial type, and congruence.

	Congruent^a^		Incongruent^b^		Attentional bias indices^c^	
Trial type	*M*	*SD*	*M*	*SD*	*M*	*SD*
House Goal Phases						
Angry faces vs. houses	441	70	437	74	-4	35
Angry faces vs. control	423	67	441	71	18	30
Houses vs. control	438	66	452	58	14	35
Angry Face Phases						
Angry faces vs. houses	418	68	456	69	38	33
Angry faces vs. control	419	66	450	66	31	32
Houses vs. control	440	66	435	66	-6	30

^a^Congruent refers to trials in which the probe replaced the image category first mentioned under trial type.

^b^Incongruent refers to trials in which the probe replaced the image category mentioned second under trial type.

^c^Attentional bias indices were calculated by subtracting RTs on congruent trials from RTs on incongruent trials.

We performed two separate ANOVAs. The first ANOVA was performed on the two trial types involving angry faces (angry face versus houses; angry face versus control car stimuli) to address our main hypotheses of the present study. We also performed another ANOVA on trials involving houses and control stimuli (car) to ensure that house images attracted attention over control stimuli selectively in the house goal condition. We ran separate ANOVAS because we varied the stimuli shown on each trial type [23, 24].

### Analyses on all trials involving angry faces

**Fig 2:**
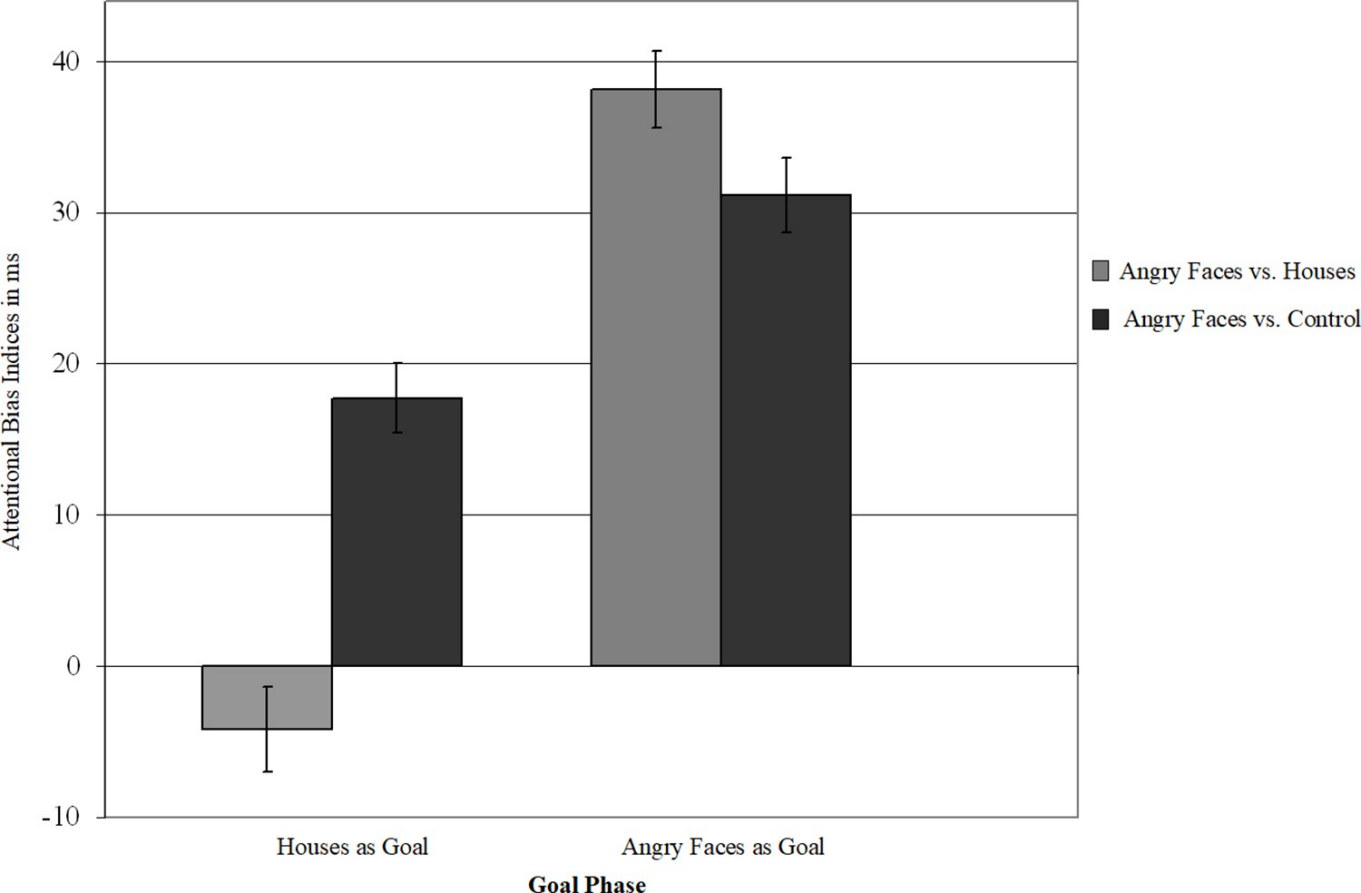
Attentional bias indices for the two trial types presenting angry faces. Angry faces were presented in comparison to houses or control car stimuli in house goal and angry face goal phases. Positive attentional bias indices indicate attention towards angry faces, negative attentional bias indices attention towards houses or control car stimuli. Error bars represent standard errors of mean.

For all trials presenting angry faces, congruency was determined with respect to the angry face (congruent means the probe shared the location of angry face cues). For the first ANOVA, congruency to the angry face (congruent, incongruent), comparison stimulus (houses, control), and goal phase (angry faces relevant, houses relevant) were within factors and goal order (angry face goal first, house goal first) between factor (see Fig 2). This ANOVA allowed us to test whether angry faces evoke an attentional bias (e.g., indicated by a main effect of congruency) and whether the nature of the goal and/ or the comparison stimuli modifies attention allocation to angry faces (e.g., interactions between congruency and comparison stimulus or goal phase). We used goal order (i.e., the goal with which participants started) to make sure that this factor did not bias the results.

Analyses revealed a significant main effect of angry face congruency, *F*(1,169) = 182.79, *p* < .001, ƞ^2^p = 0.52, reflecting an attentional bias to angry faces (*M* = 21 ms, *SD* = 20 ms). Further, there was a significant main effect of comparison stimulus, *F*(1,169) = 15.03, *p* < .001, ƞ^2^p = 0.08, and a significant interaction between goal phase and comparison stimulus, *F*(1,169) = 5.38, *p* < .023, ƞ^2^ p = 0.03.

Of greater theoretical importance were the significant interactions between angry face congruency and goal phase, *F*(1,169) = 121.07, *p* < .001, ƞ^2^p = 0.42, reflecting a larger attentional bias to angry faces when angry faces were goal relevant, *M* = 35 ms; *SD* = 28 ms, compared to when the background goal made houses goal relevant, *M* = 7 ms; *SD* = 24 ms, *t*(170) = 10.96, *p* < .001, 95% CI [22.85, 32.89]. Both biases were significantly different from zero, *t*(170) = 16.45, *p* < .001, 95% CI [30.50, 38.81], and *t*(170) = 3.64, *p* < .001, 95% CI [3.10, 10.46], respectively, indicating that angry faces still attracted attention when houses were relevant.

The interaction between comparison stimulus and angry face congruency was also significant, *F*(1,169) = 182.79, *p* < .001, ƞ^2^p = 0.52, indicating a larger attentional bias to angry faces in the presence of control stimuli (cars), *M* = 24 ms; *SD* = 24 ms, than in the presence of house cues, *M* = 17 ms; *SD* = 25 ms, *t*(170) = 3.38, *p* < .002, 95% CI [3.10, 11.80]. However, both attentional bias scores to angry faces in the presence houses and in the presence of control car stimuli were significantly different from zero, *t*(170) = 13.15, *p* < .001, 95% CI [20.77, 28.12], and *t*(170) = 8.90, *p* < .001, 95% CI [13.22, 20.76], respectively. Importantly, these interactions were qualified by an interaction between goal phase, comparison stimulus, and angry face congruency, *F*(1,169) = 41.17, *p* < .001, ƞ^2^p = 0.19. All other effects, *F*s < 3.8, *ns*.

To explore the three-way interaction, we ran an ANOVA with angry face congruency and comparison stimulus (houses, control) as within factors and the goal order (angry face goal first, house goal first) as between factor *per goal phase*. When angry faces were goal relevant, analyses revealed a significant main effect of angry face congruency, *F*(1,169) = 271.17, *p* < .001, ƞ^2^p = 0.62, reflecting an attentional bias to angry faces, *M* = 35 ms; *SD* = 28 ms. Further, there was a significant interaction between comparison stimulus and angry face congruency, *F*(1,169) = 6.85, *p* < .02, ƞ^2^p = 0.04, indicating a smaller attentional bias to angry faces in the presence of control stimuli (cars), *M* = 31 ms; *SD* = 32 ms, than in the presence of house cues, *M* = 38 ms; *SD* = 33 ms, *t*(170) = -2.60, *p* < .02, 95% CI [-12.32, -1.69]. Both biases were significantly different from zero, *t*(170) = 12.74, *p* < .001, 95% CI [26.33, 35.98] and *t*(170) = 14.94, *p* < .001, 95% CI [33.12, 43.20], respectively. All other effects, *F*s < 1.07, *ns*.

When the background goal made houses goal relevant, we found a significant main effect of angry face congruency, *F*(1,169) = 13.12, *p* < .001, ƞ^2^p = 0.07, reflecting an attentional bias to angry faces, *M* = 7 ms; *SD* = 24 ms. Further there was a significant interaction between comparison stimulus and angry face congruency, *F*(1,169) = 37.84, *p* < .001, ƞ^2^p = 0.18, indicating more attention to angry faces in the presence of control stimuli (cars), *M* = 17 ms; *SD* = 30 ms, than in the presence of house cues, *M* = -4 ms, *SD* = 37 ms, *t*(170) = -6.17, *p* < 0.001, 95% CI [-28.92, -14.90]. The attentional bias to angry faces in the presence of control stimuli was significantly different from zero, *t*(170) = 7.63, *p* < .001, 95% CI [13.15, 22.32]. Thus, in line with our prediction, even in the presence of neutral goals, angry faces were preferentially attended when they were not paired with goal-relevant stimuli. In contrast, the bias score was not significantly different from zero in the presence of houses, *t*(170) = -1.49, *p* = .138, 95% CI [-9.70, 1.36], suggesting that attention was not preferentially allocated to either angry faces or house cues when angry faces were paired with neutral but goal-relevant stimuli. Using a median RT treatment, the attentional bias towards houses in house goal phases was significant, *M* = -8 ms, *SD* = 36 ms, *t*(170) = -2.83, *p* < .006, 95% CI [2.37, 13.23]. All other effects, *F*s < 1, *ns*, except for a significant main effect of comparison stimulus, *F*(1,169) = 22.94, *p* < .001, ƞ^2^p = 0.12.

### Analyses on trials comparing house cues to control stimuli

**Fig 3:**
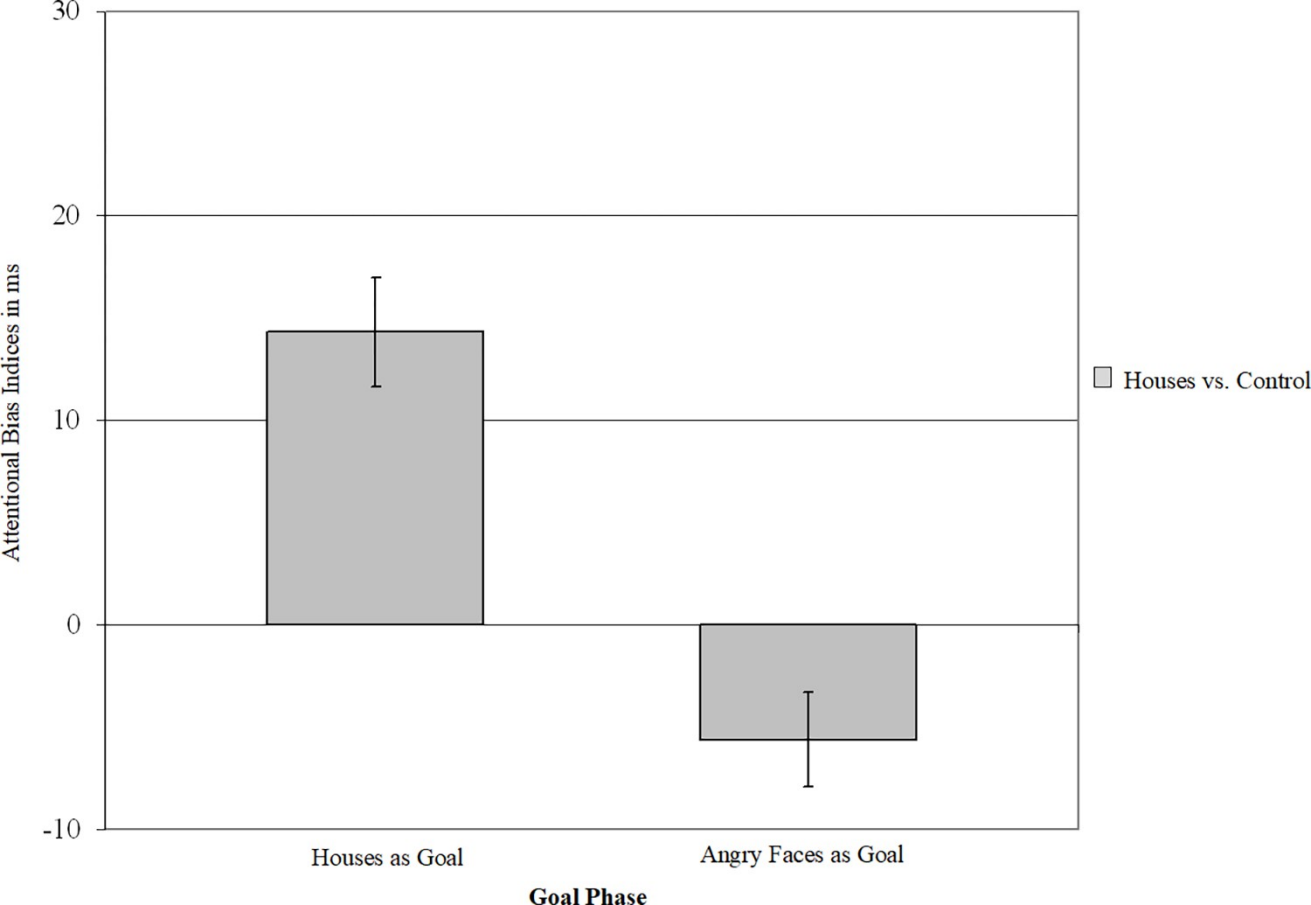
Attentional bias indices for trials presenting houses. Houses were presented in comparison to control car stimuli in house goal and angry face goal phases. Positive attentional bias indices indicate attention towards houses, negative attentional bias indices attention towards control car stimuli. Error bars represent standard errors of mean.

A final ANOVA on trials presenting house images together with control images was performed using goal phase (angry faces as goal, houses as goal), house congruency (congruency of probe location and house cue location; congruent, incongruent) as within factors and goal order (angry faces as first goal, houses as first goal) as between factor (see Fig 3). This ANOVA allowed us to test whether houses evoke an attentional bias (e.g., main effect of congruency) and whether this depends on whether houses are goal relevant (e.g., interaction between congruency and goal phase). The analyses revealed a significant main effect of goal phase, *F*(1,169) = 12.48, *p* < .002, ƞ^2^p = 0.07, and an interaction between goal order and goal phase, *F*(1,169) = 11.18, *p* < .002, ƞ^2^p = 0.06. More importantly, we found a main effect of house congruency, *F*(1,169) = 5.59, *p* < .020, ƞ^2^p = 0.03, reflecting an attentional bias towards houses across phases (*M* = 4 ms, *SD* = 25 ms) in the presence of control stimuli (cars). This was qualified by an interaction between goal phase and goal congruency, *F*(1,169) = 37.67, *p* < .001, ƞ^2^p = 0.18, reflecting a significantly higher attentional bias to houses in house goal phases, (*M* = 14 ms, *SD* = 35 ms), than in phases with an angry goal (*M* = -5 ms, *SD* = 30 ms), *t*(170) = -6.14, *p* < .001, 95% CI [-26.01, -13.35]. Further, whereas the attentional bias to houses in house goal phases was significantly different from zero indicating attention to house cues, *t*(170) = 5.38, *p* < .001, 95% CI [9.05, 19.55], the attentional bias to houses in the angry faces goal phases differed significantly from zero, *t*(171) = -2.45, *p* < .016, CI [-10.13, -1.09] and indicated attentional avoidance of houses in this phase.

To understand whether the reduction in attention bias from goal-relevant to goal-irrelevant phases differed for houses and angry faces, we subtracted the attentional bias score to houses/angry faces for goal irrelevant phases from the attentional bias score to houses/angry faces in goal relevant phases. The reduction in attentional bias for house cues (*M* = -20 ms, *SD* = 42 ms) was not significantly larger than for angry face cues (*M* = -13 ms, *SD* = 39 ms), *t*(170) = -1.48, *p* = .140, 95% CI [-14.58, 2.07]. All other effects, *F*s < 2.74.

### Social anxiety

To test the effect of social anxiety on attention to angry faces, we performed an ANCOVA on the two trial types involving angry faces (angry face versus house; angry face versus control stimuli) with angry face congruency, comparison stimulus (houses, control), and goal phase (angry faces relevant, houses relevant) as within factors, the goal order (angry face goal first, house goal first) as between factor, and the SPIN score as covariate. We still found a significant main effect of angry face congruency, *F*(1,168) = 4.66, *p* < .04, ƞ^2^p = 0.03. Importantly, the analyses also revealed an interaction between the SPIN score and angry face congruency, *F*(1,168) = 4.66, *p* < .04, ƞ^2^p = 0.03, reflecting more attention to angry faces with higher levels of social phobia, *r* = .164, *p* < .04. All other effects were not significant, *F*s < 3.70, *ns*, except for the interaction between goal phase, comparison stimulus, and angry face congruency, *F*(1,168) = 8.83, *p* < .004, ƞ^2^p = 0.05. Further, the interaction between angry face congruency, goal phase, and SPIN score approached significance, *F*(1,168) = 3.03, *p* = .084, ƞ^2^p = 0.02.

**Fig 4:**
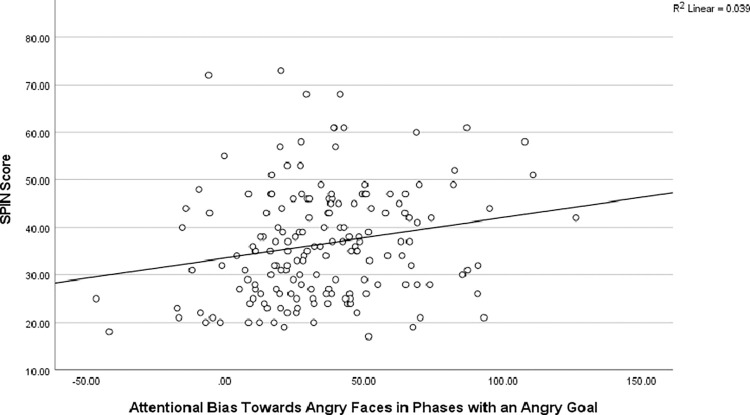
Association between social anxiety and attention to angry faces. Scatterplot showing the association between the SPIN score (y-axis) and attention bias to angry faces across the trials showing angry faces (in ms, x-axis) in the phases where angry phases were goal relevant.

To understand how social phobia impacts attention to angry faces depending on the background goal, we ran ANCOVAs with angry face congruency and comparison stimulus (houses, control (cars)) as within factors, the goal order (angry face goal first, house goal first) as between factor, and the SPIN score as covariate *per goal phase*. When angry faces were goal relevant, analyses revealed a significant main effect of angry face congruency, *F*(1,168) = 7.04, *p* < .01, ƞ^2^p = 0.04, and an interaction between the SPIN score and angry face congruency, *F*(1,168) = 6.98, *p* < .01, ƞ^2^p = 0.04, reflecting more attention to angry faces with higher levels of social phobia, *r* = .198, *p* < .01, see also Fig 4. All other effects, *F*s < 3.3, *ns*.

When the background goal made houses goal relevant, there were no significant effects or interactions, either with or without the SPIN score, *F*s < 2.5, *ns*, except for the significant interaction between comparison stimulus and angry face congruency, *F*(1,168) = 5.70, *p* < .02, ƞ^2^p = 0.03, that indicates more attention to angry faces in the presence of control stimuli than in the presence of houses. Importantly, the main effect of angry face congruency was not any more significant, *F*s < 1, *ns*.

We did not find any significant interactions with the other measures of anxiety and emotion, except for interactions with the BFNE approaching significance, mirroring the effects of the SPIN. In a linear regression using the attentional bias score towards angry faces (when angry faces were goal relevant) as dependent variable and the scores from all questionnaires as predictors, the SPIN score is the only significant predictor, *B* = .624, *t* = 2.74, *p* < .01. All other *p*s > .384, *ns*.

## General discussion

The present study investigated attention to angry faces and a neutral stimulus category, (houses), whilst varying whether houses or angry faces were relevant to a goal pursued in a secondary task. When angry faces were relevant to this background goal, participants attended to angry faces both in the presence of houses and control stimuli (cars). Heightened levels of social phobia enhanced the attentional bias to angry faces when angry faces were goal relevant. However, when houses were goal relevant, attention to angry faces was attenuated. Specifically, on trials comparing angry faces to houses, attention to angry faces was absent. On trials comparing angry faces to control stimuli, we still found an attentional bias to angry faces, but it was significantly attenuated relative to when angry faces were goal relevant. There was no evidence that social anxiety enhanced attentional bias to angry faces when houses were goal relevant. Finally, when houses were goal relevant, there was evidence for attention bias to houses on trials comparing houses to control car stimuli.

Our study provides the first evidence that social phobia enhances the effects of threat-related top-down goals [cf. 14]. Previous studies failed to find evidence for this assumption [e.g., 27, 31; see also 38]. However, in these studies, participants were instructed to search for or to actively attend towards threatening stimuli in the attention task. Such manipulations evoke a strong attentional bias to threat in all participants, leaving little sensitivity for detecting effects of anxiety. In contrast, in our study, we induced the threat-related goal outside of the attention task, which allowed us to activate a goal that was irrelevant to the attention task, providing greater sensitivity for detection of individual differences within the attention task. It is not clear what mechanism underpins the enhancement of attentional bias to angry faces when angry faces were goal relevant in social anxiety. One possibility is that threat-related goals in the present study were more motivating to socially anxious observers [14]; the findings align with previous research showing that motivation, such as higher reward, enhances attention [16]. However, several other explanations exist. For example, it may be easier for participants high in social anxiety to adopt a threat-relevant goal because it aligns with existing or recently activated goals. Further, future research must explore whether a threat-related goal would generalize and cause attentional bias to other threatening stimuli than the goal-relevant stimuli (e.g., would generalize from threatening animals to angry faces).

In phases where houses were relevant, we found reduced attention to angry faces, especially when the comparison stimuli were goal relevant (houses; cf. [51]). Though an attention bias to angry faces was still apparent when angry faces were paired with control stimuli (cars), showing that the house goal did not entirely suppress the bias toward angry faces in the same way as the angry face goal did for bias toward houses. In fact, when angry faces were the goal, there was attentional avoidance of houses relative to the control condition. This result is in keeping with the idea that threat-relevant stimuli have special status within attentional system [4]. However, the level of reduction in bias to both, angry faces, and houses, depending on their goal relevance is the same. Further, we cannot exclude the possibility that the results for angry faces are due to them being a social stimulus, rather than their threat value. Future research should therefore include neutral faces as control stimuli in the attention task. This was not included in our paradigm due to the number of trials this would have required. The fact that we find effects of social anxiety on attention to angry faces suggests that our findings are due to the threat value of the faces. However, we cannot exclude the possibility that social anxiety would have been associated with an attentional bias also to neutral faces, for instance, because for a socially anxious observer neutral expressions are threatening.

Importantly, our results indicate that a neutral background goal can override attention to angry faces, even in individuals high in social anxiety. This finding adds to a growing body of evidence suggesting that an attentional bias to neutral, non-emotional stimuli can be evoked by manipulation of short-term goals, despite these goals having no inborn or overlearned emotional or motivational significance [8, 23, 30]. This suggests that the attentional system is much more flexible than previously assumed and does not require the presence of a threat detector to produce a rapid attentional bias [2]. Indeed, even where social anxiety enhances attention bias following the activation of threat goals, this does not necessarily imply that biases are automatic or that they reflect the operations of an oversensitive threat evaluation systems outside of people’s control (see [52] for an overview of the different models of attentional bias in anxiety). The same result could be driven by goals, such as a goal to look out for signs of negative evaluation or social mishaps to implement safety behaviours or avoidance [53], or other top-down settings [54].

Our research is not without limitations. For instance, the inclusion of a baseline measure or control group without any goal would have allowed us to determine whether making angry faces goal relevant enhanced attentional bias to the angry faces. This was not included in our paradigm to keep the overall length of the task manageable for participants. Nevertheless, without this condition it is not clear exactly how the goals affected attention bias to angry faces in the context of no goal manipulation. Additional areas for exploration in future research are also recommended. First, it will be of relevance to explore the nature of any underlying goals associated with anxiety that may explain preferential attention to threat, and to begin to consider how these goals might be corrected [55]. Recent research for example, has used contextual goals as a route to modify attentional bias [56]. Further, extensions of these findings to clinical samples will also enhance our understanding of the relevance of goals for threat-relevant attention biases in clinical anxiety.

## Conclusion

In sum, our research suggests that contextual factors such as recently activated goals and the nature of distractor stimuli impact attentional bias to threat in addition to individual differences in social phobias. The results indicate that attention may be preferentially allocated to threat stimuli but that this can be overridden by short-term goals. We hope that future research will use this approach to better understand and treat (mal)adaptive biases, for instance, by considering variations in background goals.
